# Dual Burden: Osteomyelitis Secondary to Disseminated Coccidioidomycosis in an Immunocompetent Patient

**DOI:** 10.7759/cureus.102267

**Published:** 2026-01-25

**Authors:** Carlos Gonzalez, Omar Abdelkarim, Bailey Mirmelli, Jose Mojardin, Sonal Haerter, Moustafa Hazin

**Affiliations:** 1 Internal Medicine, Creighton University School of Medicine, Phoenix, USA

**Keywords:** cocci, fungal osteomyelitis, invasive fungal infections, invasive fungus, osteo-myelitis

## Abstract

Disseminated coccidioidomycosis is a rare but serious complication of infection with *Coccidioides *species that can affect multiple organ systems, including bone. We report the case of an immunocompetent adult who developed disseminated coccidioidomycosis complicated by osteomyelitis. The patient initially presented with back pain and foot findings suggestive of infection. Diagnostic workup, including imaging and microbiologic studies, confirmed *Coccidioides *infection with osseous involvement. The patient was managed with prolonged antifungal therapy and multidisciplinary care.

## Introduction

*Coccidioides immitis *(*C. immitis*) is a dimorphic soil-dwelling fungus endemic to arid regions of the Western hemisphere, including the southwestern United States, Mexico, Central America, and South America [1,2]. Infection occurs through inhalation of aerosolized arthrospores, which lodge in the alveoli and undergo transformation into spherules filled with endospores [3]. Most infected individuals remain asymptomatic or develop a self-limited pulmonary illness characterized by fever, cough, dyspnea, chest pain, weight loss, myalgias, or maculopapular rash [1]. Although most cases are mild, a small subset of patients progresses to disseminated disease, which most commonly involves the skin, bones, central nervous system, including vertebral osteomyelitis and meningitis [1,4]. Musculoskeletal dissemination is particularly uncommon, occurring in 0.5% to 1% of infected individuals [5]. Risk factors associated with dissemination include immunocompromised status, pregnancy, male sex, age over 60 years, discrete genetic factors, and Black or Filipino race [6,7]. 

The initial test for coccidioidomycosis diagnosis often involves serology, such as complement fixation or tube precipitin assays, but *Coccidioides *can also be cultured or seen in microscopy [1,8]. Patients with suspected coccidioidomycosis should obtain chest imaging and blood tests. Lung biopsy can be considered in patients with peripheral solitary pulmonary nodules or if the diagnosis cannot be established by methods such as those described previously [9]. For disseminated coccidiomycosis, additional diagnostic tests, including MRI and lumbar punctures, can be obtained depending on clinical presentation [9]. 

Treatment depends on disease severity and extent, with azole antifungals such as fluconazole serving as the first-line therapy for typical coccidioidomycosis, often for prolonged cases of three to six months or longer [1]. However, disseminated coccidioidomycosis treatment is more involved, particularly with osseous involvement, and patients can expect to undergo at least one year of treatment with antifungals, including IV amphotericin B in severe cases [10]. Given the rarity of disseminated musculoskeletal disease, particularly in the context of immunocompetent individuals, we report a case of osteomyelitis secondary to disseminated coccidioidomycosis in an otherwise immunocompetent patient. 

## Case presentation

A 50-year-old woman of Indigenous Mexican descent was hospitalized for 14 days at a tertiary care hospital in the southwestern United States in April 2024, after which her care was assumed by their trauma service. In April 2025, she was transferred to our primary care clinic for ongoing management. Communication was facilitated through her Spanish-fluent daughter and a certified medical interpreter. Her past medical history was limited but notable for borderline hypertriglyceridemia without ongoing treatment. She denied tobacco, alcohol, and illicit drug use and reported no known drug allergies. 

The patient first developed worsening back pain accompanied by skin lesions on her back, as well as swelling, erythema, and pain in her left foot. Initial evaluation and MRI in April 2024 revealed multiple abscesses at the iliac crest with associated bony destruction, osteomyelitis of the bilateral posterior iliac crests, and a right iliacus muscle abscess. There was also a left navicular and medial distal tibia subcutaneous abscess, suspicious for osteomyelitis with median cuneiform involvement on its posterior aspect. Cutaneous findings included a phlegmonous tissue along the proximal dorsum of the foot, medial aspect just beyond the ankle joint, overlying the navicular. She also had anemia. She was diagnosed with osteomyelitis involving the spine and left foot, as well as disseminated coccidioidomycosis per enzyme immunoassay. Initial blood cultures from hospital day 1 yielded no growth. 

She subsequently underwent incision and drainage of the left foot on hospital day 3, with wound cultures obtained the following day growing *Coccidioides* spp. At that time, a nonspecific left lung infiltrate was identified on serial chest imaging, thought to be secondary to pulmonary coccidioidomycosis. Repeat foot wound cultures obtained on hospital days 6 and 11 showed no growth, while a coccidioidomycosis enzyme immunoassay performed on hospital day 7 was positive for both IgM and IgG antibodies. Once clinically stabilized, she was discharged on a four-week course of twice-daily oral doxycycline (100 mg) and cefuroxime (500 mg), in addition to indefinite daily oral fluconazole therapy (400 mg). 

**Figure 1:**
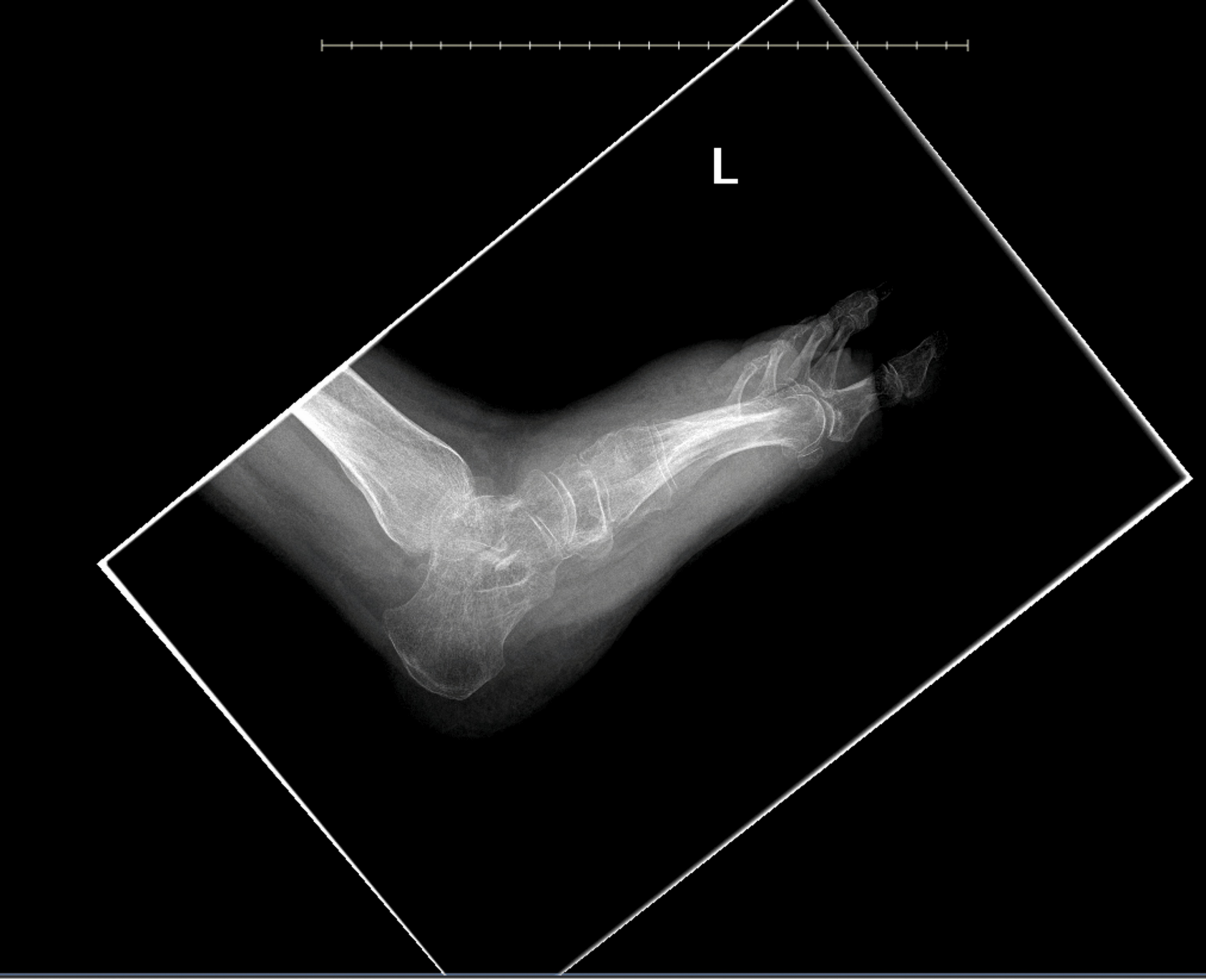
Left foot imaging from February 2025 Image impression shows no acute fracture or dislocation. Chronic fracture third metatarsal base. Osseous demineralization. No radiographic evidence of osteomyelitis. A large amount of soft tissue swelling

Post-discharge, the patient continued follow-up with the hospital trauma service for wound care, including Biostep and silver sulfadiazine 1% applied every three days. Gradual wound improvement was observed. Follow-up imaging in February 2025 showed no radiographic signs of osteomyelitis with a large amount of soft tissue swelling (Figure 1). She also continued regular follow-up with the infectious disease service for monitoring and management of disseminated coccidioidomycosis with subsequent titers. 

**Figure 2:**
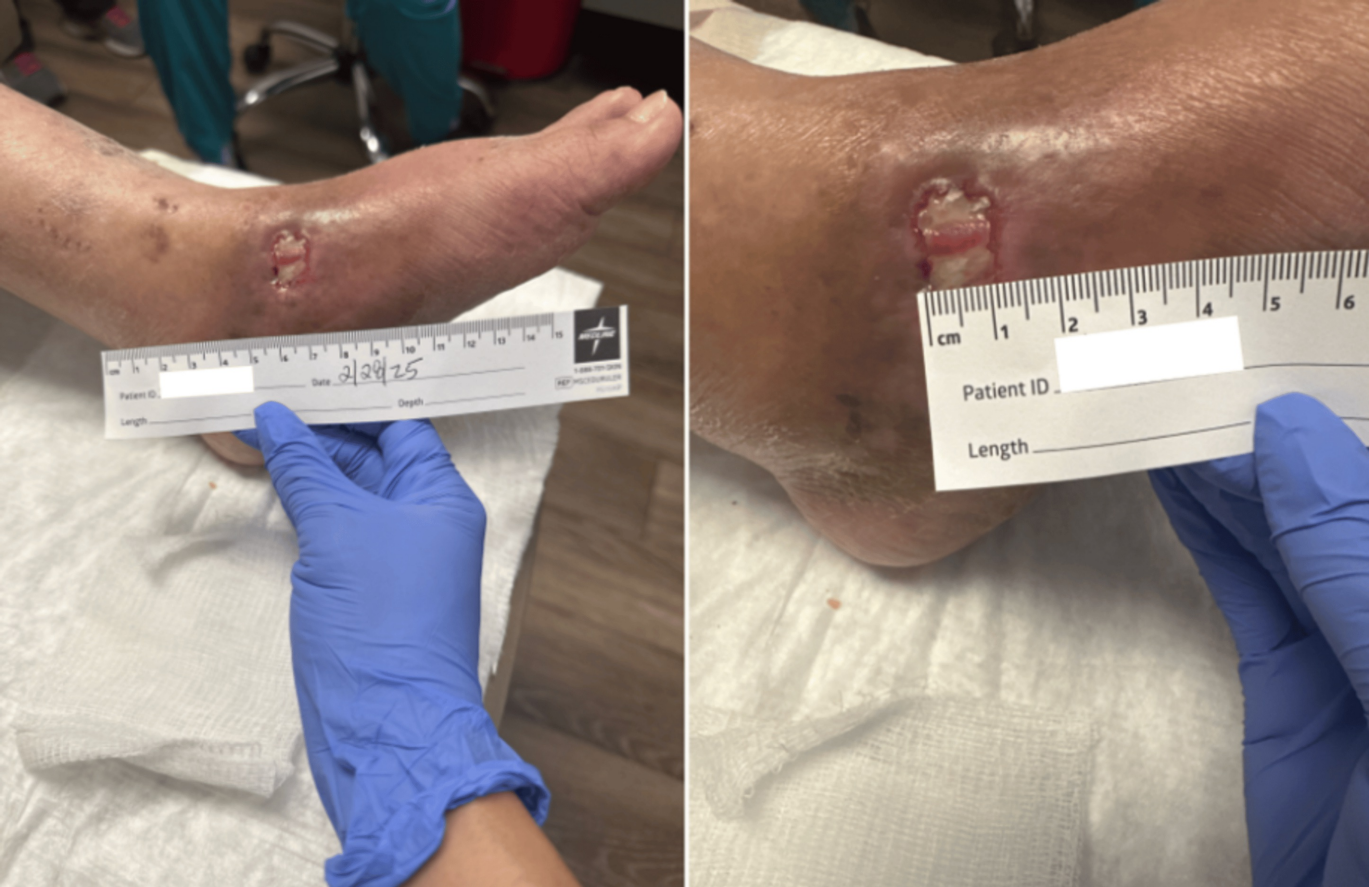
Left foot wound on February 2025 Clinical photographs of the patient’s left foot wound showing a full-thickness ulcer with a beefy red wound bed and surrounding erythema. The wound was measured to be 1.2 cm in length

**Figure 3:**
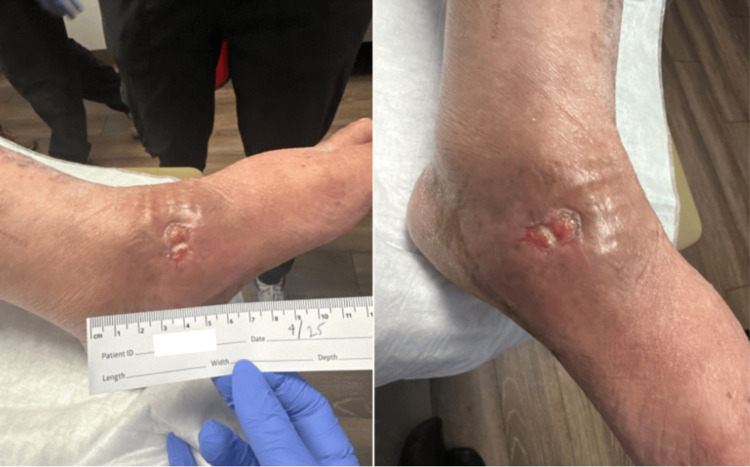
Left foot wound on April 2025 Clinical photographs showing interval improvement of the left foot ulcer compared with Figure 1. The wound measured approximately 1 cm in diameter with decreased surrounding erythema, consistent with a progressive healing ulcer, continued wound care, and antifungal therapy

Upon arriving at our clinic in May 2025, she reported persistent tenderness throughout her left foot, which worsened with wound manipulation and was managed with over-the-counter ibuprofen. She denied systemic symptoms such as fever, chills, or night sweats. Physical examination demonstrated a left foot wound measuring 1.5 cm in length and 2.0 cm in width. Drainage was moderate and serosanguineous, and the wound bed appeared beefy red (Figure 2). The left leg was warm to the touch, with significant edema and diffuse tenderness to palpation. Pulses were palpable and symmetric in both the dorsalis pedis and posterior tibial arteries. Sensation was intact. Laboratory results obtained at presentation are shown in Table 1. A follow-up presentation is shown in Figure 3. 

**Table 1:** Laboratory data WBC: white blood cells; RBC: red blood cells; MCV: mean corpuscular volume; MCH: mean corpuscular hemoglobin; MCHC: mean corpuscular hemoglobin concentration; RDW: red-cell distribution width; ABS: absolute; CBC: complete blood count; MPV: mean platelet volume; CF: complement fixation; ELISA: enzyme-linked immunosorbent assay; ESR: erythrocyte sedimentation rate; CRP: C-reactive protein

Parameter (unit)	Lab value	Reference range
WBC (thousand/uL)	5.9	3.6-11.1
RBC (million/uL)	4.58	3.69-5.19
Hemoglobin (gm/dL)	12.1	11.4-14.4
Hematocrit (%)	36.8	33.3-45.8
MCV (fL)	80	79-99
MCH (pg)	26.3	28.0-32.0
MCHC (gm/dL)	32.8	32.0-36.0
RDW (%)	18.0	11.5-14.5
Platelet count (thousand/uL)	269	150-450
Neutrophils (%)	70.6	40-70
Lymphocytes (%)	21	20-40
Monocytes (%)	6.5	2-8
Eosinophils (%)	1	1-3
Basophils (%)	0.9	0-1
ABS neutrophils (thousand/uL)	4.2	2.0-8.0
ABS lymphocytes (thousand/uL)	1.2	0.6-4.8
ABS monocytes (thousand/uL)	0.4	0.1-2.0
ABS eosinophils (thousand/uL)	0.1	0.0-0.7
ABS basophils (thousand/uL)	0.1	0.0-0.2
CBC scan	Auto dif	-
MPV (fL)	9.8	7.5-11.5
Coccidioides antibody by CF	1:64	<1:2
Antinuclear Ab, IgG by ELISA	None detected	None detected
Phosphorus (mg/dL)	3	2.4-4.7
ESR auto (mm/hr)	43	0-30
CRP high sensitivity (mg/L)	2.2	<=10.0
25-Hydroxyvitamin D3 (ng/mL)	1.2	20-50
25-Hydroxyvitamin D2 and D3 total (ng/mL)	<2.0	30.0-80.0

At a follow-up in June 2025, the patient reported that she was adherent to daily fluconazole 400 mg with no side effects. Lesions did not demonstrate any progression. Liver function tests remained within normal limits. By July 2025, wounds were healing with significantly reduced drainage and persistence of left foot edema. *Coccidioides *complement fixation titer at this time was 1:32, which dropped from 1:64 in March 2025. In August 2025, the patient reported developing a new left gluteal abscess without fevers or night sweats. In October 2025, inflammatory markers showed improvement, with C-reactive protein decreasing from 2.2 mg/dL in February 2025 to 1.7 mg/dL, and erythrocyte sedimentation rate decreasing from 43 to 34 mm/hr. The patient reported being fully compliant with therapy throughout visits. 

In November 2025, she was increased to a daily dose of 600 mg oral fluconazole. The patient reported continued clinical improvement with minimal residual drainage, and *Coccidioides *titers had decreased to 1:16, consistent with a favorable response to prolonged antifungal therapy. 

## Discussion

*C. immitis* has been identified as an organism capable of infecting the musculoskeletal system [8]. In disseminated infections, it is estimated that 10%-50% of cases involve skeletal tissue [11]. Most reported *C. immitis* osteomyelitis cases affect the axial skeleton, classically the vertebral spine [11,12]. However, past reports have described cases of lower extremity osteomyelitis in the knee and ankle [13]. Evidence suggests that *C. immitis* osteomyelitis is associated with immunosuppressed states, and few cases have documented disseminated *C. immitis* infection in immunocompetent patients [12-14]. 

The patient’s positive *Coccidioides *antibody titer confirms exposure and supports active infection, while the elevated erythrocyte sedimentation rate reflects an ongoing inflammatory process consistent with both disseminated coccidioidomycosis and osteomyelitis [15]. Her low 25-hydroxyvitamin D2 and D3 levels may reflect chronic illness, nutritional deficiency, or impaired absorption [16]. However, although vitamin D deficiency has been associated with extrapulmonary disease in other granulomatous intracellular infections such as tuberculosis, the relevance of vitamin D status to disease severity or extrapulmonary dissemination in coccidioidomycosis has not been directly studied [17]. Although specific evidence for fungal pathogens is limited, vitamin D is known to modulate innate immunity through the regulation of antimicrobial peptides (e.g., cathelicidin/LL-37) and autophagy, mechanisms crucial for controlling intracellular pathogens like *Mycobacterium tuberculosis* [18]. All other laboratory findings were within normal limits, underscoring the atypical presentation of disseminated coccidioidomycosis in this immunocompetent patient. 

Regarding the treatment of *C. immitis* osteomyelitis, reported approaches have included oral azole therapy and surgical debridement [19,20]. Fluconazole and itraconazole are most used, whereas IV amphotericin B is generally avoided due to toxicity, though some cases have employed combination therapy with liposomal amphotericin B followed by step-down to oral fluconazole after approximately one month [20]. Surgical debridement has been performed in select cases to reduce pathogen burden and remove necrotic tissue. However, depending on the patient’s immune status, these interventions may not be sufficient [21]. In the present case, the patient underwent surgical debridement and received four weeks of antibiotic therapy in addition to oral fluconazole. At follow-up, her wound demonstrated gradual improvement. 

One possible contributing factor to the development of disseminated *C. immitis* infection in this immunocompetent individual may have been a delayed diagnosis, potentially related to the patient’s uninsured status and cultural or language barriers. The language barrier is well-documented in the literature and raises important questions about how such delays can be prevented in future patients [22,23]. Although healthcare institutions are legally required to provide linguistic services, the quality and consistency of these services vary widely in practice [24]. Ensuring access to trained medical interpreters, rather than relying on family members or informal community translators, can help build trust between patients and providers and improve both early recognition of illness and adherence to treatment, particularly for complex diseases. 

Additionally, the patient's lack of medical insurance likely played a major role in delaying diagnosis and treatment. Uninsured patients have limited access to appropriate screening and are less likely to seek medical care as symptoms arise [25,26]. As a result, the advanced extent of this patient’s disease may reflect the consequences of delayed or insufficient medical attention. By the time she presented to the hospital, the infection had progressed from the pelvis to the foot. Although asymptomatic dissemination has been reported in immunocompetent individuals, coccidioidal osteomyelitis typically demonstrates a slow, chronic course [27]. Earlier recognition and intervention may have mitigated the severity of disease progression in this case. Engaging patients in discussions about barriers to care may help clinicians identify and address factors that contribute to delays in diagnosis and treatment. 

## Conclusions

The development of advanced osteomyelitis from disseminated *C. immitis* infection in an immunocompetent patient raises concern for possible contributing factors such as vitamin D deficiency, diagnostic challenges, delays in diagnosis due to communication barriers, and limited access to timely care. As a result of disease progression, the patient will require lifelong antifungal therapy, underscoring the long-term consequences of delayed recognition. Overall, this case exemplifies the importance of early recognition, equitable access to care, and culturally and linguistically responsive clinical practice in preventing severe disease progression in vulnerable patients. It also highlights the need for a high index of suspicion for disseminated fungal infections in apparently immunocompetent individuals. 
